# Neurobehavioral effects of transportation noise in primary schoolchildren: a cross-sectional study

**DOI:** 10.1186/1476-069X-9-25

**Published:** 2010-06-01

**Authors:** Elise van Kempen, Irene van Kamp, Erik Lebret, Jan Lammers, Harry Emmen, Stephen Stansfeld

**Affiliations:** 1 National Institute for Public Health and the Environment, Centre for Environmental Health Research, Bilthoven, The Netherlands; 2 TNO Quality of Life, Zeist, The Netherlands; 3 Barts and the London, Queen Mary, University of London, London, UK

## Abstract

**Background:**

Due to shortcomings in the design, no source-specific exposure-effect relations are as yet available describing the effects of noise on children's cognitive performance. This paper reports on a study investigating the effects of aircraft and road traffic noise exposure on the cognitive performance of primary schoolchildren in both the home and the school setting.

**Methods:**

Participants were 553 children (age 9-11 years) attending 24 primary schools around Schiphol Amsterdam Airport. Cognitive performance was measured by the Neurobehavioral Evaluation System (NES), and a set of paper-and-pencil tests. Multilevel regression analyses were applied to estimate the association between noise exposure and cognitive performance, accounting for demographic and school related confounders.

**Results:**

Effects of school noise exposure were observed in the more difficult parts of the Switching Attention Test (SAT): children attending schools with higher road or aircraft noise levels made significantly more errors. The correlational pattern and factor structure of the data indicate that the coherence between the neurobehavioral tests and paper-and-pencil tests is high.

**Conclusions:**

Based on this study and previous scientific literature it can be concluded that performance on simple tasks is less susceptible to the effects of noise than performance on more complex tasks.

## Background

Transportation is an activity that is responsible for a large and growing proportion of environment and health effects in Europe. Noise is generally perceived as one of the problems associated with transportation. It has been estimated that approximately 20 percent of the European Union's population is exposed to road traffic noise at levels exceeding 65 dB(A) during daytime; more than 30 percent is exposed to levels exceeding 55 dB(A) during night-time [1]. Despite numerous measures in the field of noise abatement at European, national and local levels, the noise-level has not decreased. Without additional measures, more people will be exposed to higher sound levels in future decades [2]. Since noise is one of the environmental stressors purported to have adverse effects on human health and well-being [3], the noise-related disease burden is expected to rise [4].

This paper focuses on the effects of noise on primary schoolchildren. Children are suspected of being more susceptible to noise exposure for a number of reasons: since they spend their time at other settings and because they behave differently, children's exposure can differ from adults' exposure; children often cannot escape from exposure, where adults can; and children have not (fully) developed coping mechanisms and cannot always change their situation, whereas adults may have the power and/or resources to do so. These factors combine to generate or trigger a wide range of negative effects [5,6].

Among children, the effects of noise exposure on cognitive functioning were studied the most. During the last 30 years, a number of studies have investigated the effects of long-term exposure to air-, rail-, and road traffic noise among primary schoolchildren. Cognitive effects were found on reading, attention, problem solving and memory [7-23]. At the moment there is no theory that can adequately account for the circumstances in which noise will affect children's cognitive performance. In the literature, several mechanisms have been reported: e.g. the disturbance of the intelligibility of speech (irrelevant sound effect), the allocation of attention and the 'tuning-out' hypothesis. Because most of these mechanisms have in common that they are important for child's language acquisition, they are suspected to play a role especially in relation to the effects on reading [7,12,24].

From the observational studies that have investigated the effects of long-term transportation noise exposure on the cognitive functioning of primary schoolchildren (see also Additional file 1, table S1), several methodological problems emerge: firstly, these studies were usually focused on school noise exposure. However, time-activity studies show that children spend a large part of their time at home, sleeping [25,26]. From the literature, it is known that a person's sleep is important for learning and memory [27]. As a consequence it is hypothesized that the noise-related effects found in children might also be the consequence of a decrease in sleep quality, caused by night exposure at home during the night. Secondly, most of these studies have involved between-group comparisons. It is recognized that the results of these studies might be biased due to possible exposure misclassification [28]. Thirdly, it appeared that there was a lot of diversity among the cognitive tests used in these studies. Comparison shows that the same concepts were not always measured. For example, while some studies measure more technical aspects of reading (e.g. spelling and grammar) [14-16], others measure aspects of reading comprehension [17-19]. Alternatively, when administering the same reading test in different countries, cultural differences might affect the outcome. In addition, it is difficult to select appropriate tests that are sensitive to the effects of noise at specific stages of development, because of the many developmental stages through which children progress [29].

Since reliance upon insensitive developmental outcomes may cause underestimation of the effect of noise [30], it would be interesting to investigate the effects of noise on cognitive performance by means of computerized neurobehavioral tests that evaluate different aspects of central nervous system functioning in comparison with commonly used paper-and-pencil tests for reading, memory and attention. By measuring a range of neurobehavioral parameters by computerized performance tests, we build on work from the past: within the framework of the Health Impact Assessment Schiphol Airport, the feasibility of a selection of neurobehavioral tests from the Neurobehavioral Evaluation System (NES) used to investigate the effects of community noise in the school environment was tested and demonstrated. The NES, a computerized test battery, is originally developed by Baker and Letz (1986) [31] to facilitate the conduct of epidemiologic studies of populations at risk for or suffering from central nervous system dysfunction due to environmental agents [32]. The test battery is designed to assess attention, memory, learning, perceptual coding and psychomotor performance [30]. The NES was developed for adults but later adapted for use in children [33]. The feasibility study involved 159 children aged 8-12 yrs. The results of the study indicated a high level of acceptance of computerized test procedures by the children, teachers and parents and a high test-retest reliability for most tests (Pearson's r > 0.70) [30].

The primary goal of this paper was to investigate the possible relation between aircraft and road traffic noise exposure and cognitive performance in primary schoolchildren in both the home and the school setting. Since we wanted to expand the traditional paper-and-pencil tests, cognitive performance was operationalized by means of a selection of neurobehavioral tests from the Neurobehavioral Evaluation System (NES). Since less is known about the external validity of the NES towards paper-and-pencil tests that are more commonly used in studies investigating the effects of community noise on cognition, our secondary goal was to study the coherence between both type of test batteries, and to find out the added value of NES tests. The correlational pattern and factor-structure might indicate how the NES complements such paper-and-pencil tests.

For our analyses we acquired data from a Dutch sub-sample of schoolchildren living around Schiphol Amsterdam Airport that were gathered during the European 5^th ^Framework project RANCH (Road traffic and Aircraft Noise exposure and children's Cognition and Health). As part of RANCH, a cross-sectional study investigating the effects of aircraft and road traffic noise on the cognitive functioning, annoyance, and health of children attending primary schools around three airports in the United Kingdom, Spain and The Netherlands was carried out [6].

## Methods

### Selection and recruitment

**Figure 1:**
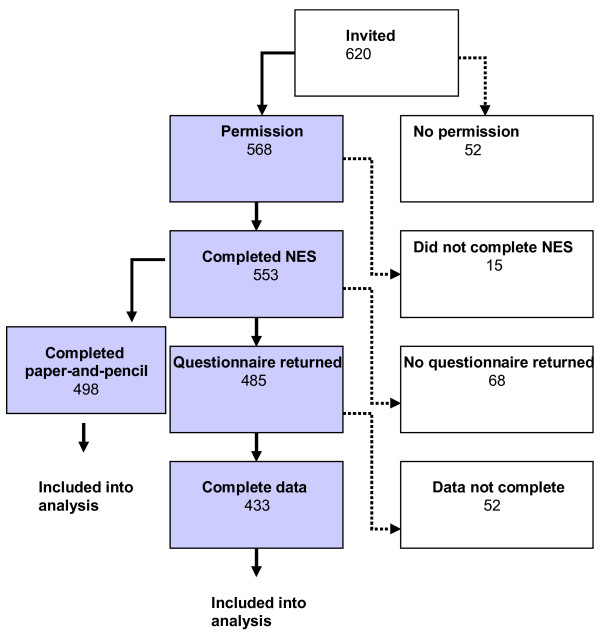
**Flowchart indicating the completeness of response and loss of information for the participating children**.

Participants were 553 primary schoolchildren that were recruited from 620 children of 24 primary schools in three Municipal Health Office areas around Schiphol Amsterdam Airport (see also figure 1). The schools were selected according to the modelled aircraft and road traffic noise exposure levels of the school area, and were matched on a neighbourhood-level indicator of property value and the percentage of non-western foreigners. Schools for children with special needs were excluded (see also [6,34]).

From the parents written consent was obtained for their children to take part in our study. Ethical approval was given by the Medical Ethics Committee of The Netherlands Organisation for Applied Scientific Research, Leiden.

### Noise exposure assessment

Noise exposure was assessed for each child by linking the school and home addresses to modelled aircraft and road traffic noise levels. The noise levels were calculated, in accordance with a standardized noise protocol, which provided a procedure for determining outdoor noise exposure. Modelled aircraft noise levels (expressed in L_Aeq 7-23 hrs_) with a resolution of 250 × 250 meter grids were obtained from nationally available noise contours from the Dutch National Aerospace Laboratory (NLR) for the year 2001. These predicted the average noise exposure from 7 to 23 hrs for a period of one year. Road traffic noise levels (expressed in L_Aeq 7-23 hrs_) were estimated from modelled composite data from 2000 and 2001, with a resolution of 25 × 25 meter grids using national standard methods [35].

### Cognitive performance

Methods used to assess cognitive functioning included selected tests from the NES and a set of paper-and-pencil tests.

### Neurobehavioral Evaluation System

**Figure 2:**
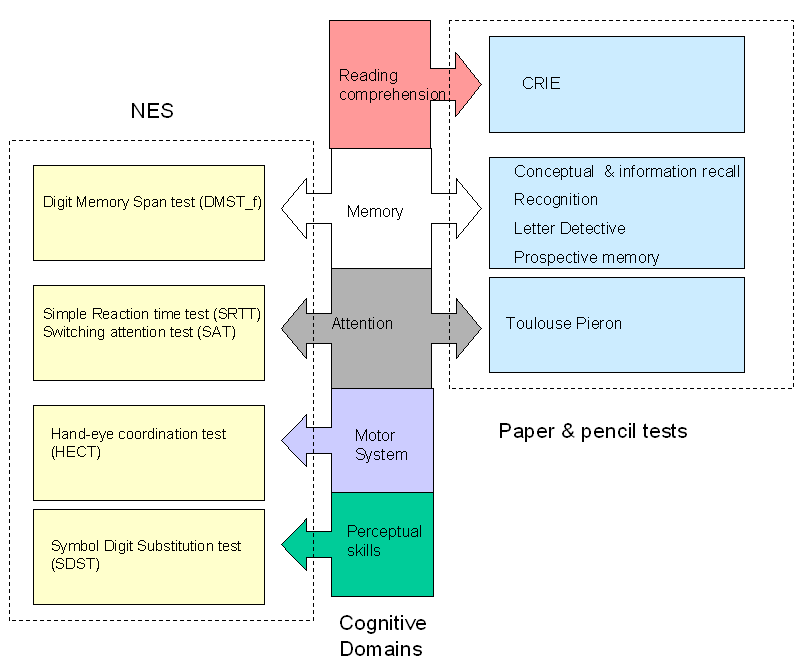
**Cognitive test batteries applied**.

The NES was administered in groups of eight children in a quiet room in school with the help of a personal computer and additional hardware (joystick/push button). The duration of the test was approximately 30 minutes. Several studies have provided data supporting the instrument's adequate psychometric properties. Evidence that the NES shows an acceptable level of reliability comes from test-retest correlations in the range 0.6 to 0.9 obtained under both laboratory and field conditions [32,33,36]. The following tests were included in the NES (see also Figure 2):

#### Simple Reaction Time Test (SRTT)

in the Simple Reaction Time Test (SRTT) the subject was asked to press a button as quickly as possible when a red square appeared on the screen. The inter-trial interval (2.5 - 5.0 sec) varied randomly to reduce effects of stimulus adaptation. Individual reaction times (in ms) were recorded.

#### Switching Attention Test (SAT)

the Switching Attention Test (SAT) was meant to test the ability of the subject to switch rapidly between responses to simple two-choice visual discriminations based on changing verbal cues. The SAT included a series of progressively challenging tasks. In the first testing condition ("Block") the subject was asked to respond to each of a series of large rectangles (which appeared on one side of the screen) presented in succession on the screen. The subject had to press the button on the corresponding side of the push button box as quickly as possible. In the second testing condition ("Arrow") the subject was asked to respond to a large arrow presented in the middle of the screen that pointed either to the left or to the right by pressing the left or right button on the button box as quickly as possible. During the final, most complex portion of the SAT ("Switch") the word "Side" or "Direction" appeared immediately before each stimulus. The stimulus was an arrow pointing either to the left or to the right, presented on either the left or right side of the screen. The subject was asked to respond to each stimulus on the basis of response criterion signified by the word presented immediately before it on each trial. The response latency and the number of switching errors were recorded.

#### Hand -Eye-Coordination Test (HECT)

in the Hand-Eye Coordination Test (HECT) the subject was asked to use a joystick to trace over a sine wave/saw tooth pattern on the computer screen. A cursor moved horizontally at a constant velocity, while the subject controlled the vertical motion of the cursor with the joystick. Deviations from the line were recorded and constituted a measure of co-ordination ability. Per trial, the vertical distance (pixels) of the cursor from the setline was sampled.

#### Symbol-Digit Substitution Test (SDST)

the Symbol-Digit Substitution Test (SDST) was a test of perceptual coding and attention. In the SDST nine symbols and nine digits were paired at the top of the screen. The subject had to press the digit keys corresponding to a test set of the nine symbols scrambled. Test measure was the time required to complete each set divided by the number of correct responses.

#### Digit Memory Span Test

in the Digit Memory Span Test (DMST) subjects had to enter into the computer progressively longer series of digits following visual presentation at a rate of one per second by the computer. After incorrectly responding to two trials at span length, the task changed such that the individuals had to enter a new digit series in reverse order. Performance was scored as the mean sequence length memorized over trials.

### Paper-and-pencil tests

The paper-and-pencil tests were administered on a separate day, during a three hour testing session under exam conditions [6]. The following paper-and-pencil tests were administered (see also Figure 2): reading comprehension was measured by the CRIE-test [37], which is a nationally standardized and normed test. In association with this test, prospective memory was measured by asking the children to write their initials in the margin when they reached two predefined points in the reading comprehension test. Episodic memory (recognition and recall) was assessed by a task adapted from the Child Memory Scale [38]. This task assessed time delayed cued recall and delayed recognition of two stories presented on a compact disc. Working memory was tested using a modified version of the Search and Memory test [39,40]. Sustained attention was measured using the Toulouse Pieron Test [41]. A more detailed description of the development and administration of the paper-and-pencil tests in RANCH can be found in Stansfeld et al (2005) [6]. Details with regard to the reliability and validity of these tests can be found in [37,41,42].

### Child and parent questionnaire

During the paper-and-pencil testing session, the children were also given a questionnaire that included questions on perceived health, perceptions of noise, annoyance, and parental support. Furthermore, the children were given a questionnaire to take home for their caregiver (preferably the mother) to complete. This questionnaire requested information on the health and behaviour of the child, noise sources heard at home, annoyance, and potential confounding factors such as glazing of the child's home, length of residency, indicators for socio-economic status, country of birth and the main language spoken at home. These variables were only available for those children whose parents also completed the questionnaire (N = 485), so parent participation served as a selection criterion for inclusion in analysis. Before data-collection, all procedures and materials were tested in a pilot study in October 2001.

### Statistical analysis

In order to investigate the reliability and the dimensionality of the cognitive tests a principal component analysis (PCA) was carried out on the scores of both the NES and the paper-and-pencil tests using SPSS for Windows (version 12.0.1). Only components that accounted for variances with Eigenvalues greater than 1 were included in this paper. To make the components more interpretable, a rotation with the Varimax method was performed resulting in components that are uncorrelated. However, in the social sciences we generally expect some correlation among factors, since behaviour is rarely partitioned into neatly packaged units that function independently of one another. As kind of sensitivity analysis an oblique rotation (with Delta = 0) was performed in addition to Varimax rotation, assuming that the resulting components may be correlated [43]. Cronbach's alphas were calculated, to test how reliable the components were in terms of internal consistency. Only children that completed both the computerized and paper-and-pencil tests were included in this part of the analysis (N = 498).

To investigate the impact of aircraft and road traffic noise on cognitive performance (operationalized by the NES), multi-level analyses were carried out using the MIXED procedure of SAS version 9.1. Multilevel modelling takes into account the hierarchical structure of the data (children grouped within schools) and enables effects at both the level of school and pupil to be included in the same model. Two-level (pupil and school) random intercept models were used. Coefficients (B) and standard errors (SE) were estimated under restricted maximum likelihood estimation (REML). Only children with complete data (N = 433) were included into the analysis. In all models, aircraft or road traffic noise exposure (at school or at home) was the main independent variable and was included as a continuous variable. The models included age (yrs), gender, main language spoken at home (Dutch/non-Dutch), long standing illness (based on parental reports of the child having either ADHD, asthma/bronchitis, eczema, epilepsy, depression, diabetes or dyslexia), parental support for school work (assessed by a self-report scale in the children's questionnaire), school glazing (single, double or triple), indicators for socio-economic status (crowding, home ownership, parental employment and mother's education), and the other noise source as potential confounders. Statistical significance of a coefficient was tested under full maximum likelihood (ML) estimation, using a Chi-square test of deviance. Further analyses were conducted, excluding children whose parents have reported that they suffered from ADHD and/or dyslexia.

## Results

Table 1 presents some general characteristics of the participating children whose parents completed a questionnaires, as well as some features of the primary schools they attend (n = 24). The average age of the children was 10 years and 6 months. Almost 49% of the sample was female and in more than 93% of the families, Dutch was the main language spoken at home. Table 2 presents the mean scores and standard deviations of the outcomes of the NES. In this table, outcomes from the feasibility study, conducted in 1997 [30] are also included. Comparison showed that the differences between the two samples fall within the range of the 95% confidence intervals.

**Table 1:** Characteristics of the children that completed the NES and whose parents returned their questionnaire (n = 485) and the schools they visit (n = 24).

	%	Mean +/- Std	Range
			

Age (yrs)		10.5 +/- 0.6	8.8 - 12.8

% girls	48.8		

			

Socio-economic status			

% employed^a)^	91.9		

% crowded^b)^	32.9		

% homeowners	81.4		

Mother's education (index 0-1)^c)^		0.5 +/- 0.3	0.0 - 1.0

			

% longstanding illness^d)^	27.7		

% ADHD	1.9		

% dyslexia	3.5		

			

% main language at home is Dutch	93.4		

			

Parental support (scale 1-12)^e)^		8.6 +/- 1.9	3 - 12

			

Glazing			

% Single glazing at school	47.8		

% Double glazing at school	49.5		

% Triple glazing at school	2.7		

% Double glazing at home	55.6		

			

Noise exposure (L_Aeq, 7-23 hr_) in dB(A)			

Aircraft noise at school		48.6 +/- 7.1^f)^	36.3 - 62.8

Aircraft noise at home		48.1 +/- 7.1^f)^	34.5 - 63.4

Road traffic noise at school		48.7 +/- 8.6^f)^	34.0 - 62.0

Road traffic noise at home		50.2 +/- 7.3^f)^	28.0 - 67.0

a) measure of the highest employment status in the child's household. At least one of the parents has to do paid work for at least 19 hrs per week.; b) This is an objective measure of the number of people per room at home. If the number of people is smaller/equal to the number of rooms than the child's household is defined as crowded; c) Mother's education was measured using a ranked index of standard qualifications. A relative index was then calculated for this variable in order to make comparisons between different measures in each country. The index ranges from 0 to 1, with higher number indicating low educational attainment; d) based on parental reports of the child having either attention deficit hyperactivity disorder (ADHD), asthma/bronchitis, eczema, epilepsy, depression, diabetes or dyslexia; e) parental support for school work is assessed by a self-report scale in the children's questionnaire This ordinal scale (alpha = 0.65) was constructed by means of the following items: "If I have a problem at school my parents/carers are ready to help." "My parents/carers are willing to come to school and talk to teachers", and "My parents/carers encourage me to do well at school". Answers were indicated on a 4-point category scale ("Never, sometimes, often, always"). The score on each of the items was summed up (possible score 3-12); f) presented are the arithmetic mean and corresponding standard deviation; Abbreviations: Std = Standard Deviation.

**Table 2:** Mean scores and variability parameters of the different NES tests for the children whose parents returned their questionnaire (n = 485) in comparison with the results of other studies.

Domain	Test/condition	RANCH				**Emmen **[29]
	
		Mean +/- Std	Min	Max	Median	Mean +/- Std
	
Attention	Simple Reaction Time					
	Latency (ms)	357 +/- 51	256	572	350	303 +/- 57

	Switching Attention					

	Errors "block" (#)	0.87 +/- 1.02	0	5	1	1 +/- 1.25

	Reaction time "block" (ms)	401 +/- 79	244	685	391	377 +/- 104

	Errors "arrow" (#)	1.71 +/- 1.59	0	8	1	1.25 +/- 1.36

	Reaction time "arrow" (ms)	557 +/- 108	245	949	546	499 +/- 95

	Errors "switch" (#)	10.52 +/- 5.70	0	27	10	10.11 +/- 5.89

	Reaction time "switch" (ms)	693 +/- 147	247	1075	700	794 +/- 203

Locomotion	Hand Eye Coordination					

	Deviation from sinus pattern (pixels)	1.76 +/- 0.43	0.77	3.13	1.77	1.97 +/- 0.32

Perceptual Coding	Symbol Digit Substitution					

	Latency (sec)	3.31 +/- 0.56	2.11	5.95	3.23	3.28 +/- 0.71

Memory	Digit Memory Span					

	Mean span-length forwards	4.78 +-/0.63	3.30	7.60	4.70	4.9 +/- 0.7

Abbreviations: ms = millisecond, # = number, sec = seconds, Std = Standard deviation, Min = minimum, Max = Maximum

The aircraft noise levels (L_Aeq, 7-23 hrs_) to which the children were exposed at school ranged from 36 to 63 dB(A); aircraft noise levels at home ranged from 34 to 63 dB(A). Aircraft noise levels were comparable with road traffic noise levels. High correlations between home and school aircraft noise levels (L_Aeq, 7-23 hrs_) were found (r > 0.9). The correlation between home and school road traffic noise levels was moderate (r ~ 0.6).

Principal Component Analysis on the two cognitive test batteries with Varimax yielded factor components with Eigenvalues greater than 1 (see also Table 3). The total percentage of variance explained by these components is 52.7%. Items of recognition, information and conceptual recall loaded highly on the first component, whereas simple reaction time and the three reaction time parameters of the switching attention test loaded highly on the second component. The items referring to the error conditions of the switching attention test loaded highly on the third component, and working memory and sustained attention loaded highly on the fourth component. The oblique rotation resulted in the same grouping of variables as the Varimax rotation and did not affect the interpretation of the components.

**Table 3:** Factor loading matrix (n = 498)*.

Test battery	Item	Factor I	Factor II	Factor III	Factor IV
NES	SRTT	0.743			

	Block_RT	0.764			

	Arrow_RT	0.769			

	Switch_RT	0.618			

	Block_f			0.631	

	Arrow_f			0.757	

	Switch_f			0.632	

	SDST			0.369	

	DMST_f				0.492

	HECT	0.538			

PP	Conceptual recall		0.922		

	Information recall		0.918		

	Recognition		0.675		

	CRIE		0.458		

	Working memory				0.682

	Sustained attention				0.608

	Prospective memory				0.469

**Factor**	**Interpretation**			**Variance explained**	Alpha**

I	Response speed and locomotion			20.3	0.76

II	Episodic memory and reading comprehension			15.1	0.77

III	Ability to switch and perceptual coding			10.8	0.58

IV	Memory and attention			6.5	0.42

**Total**				**52.7**	

* Per item only the highest loadings were presented; ** Cronbach's alpha (standardized), based on the items given in the factor loading matrix; this is a function of the item-inter-correlation and of the number of items included in the scale. Abbreviations: NES = Neurobehavioral Evaluation System; PP = Paper- and-pencil test.

**Figure 3:**
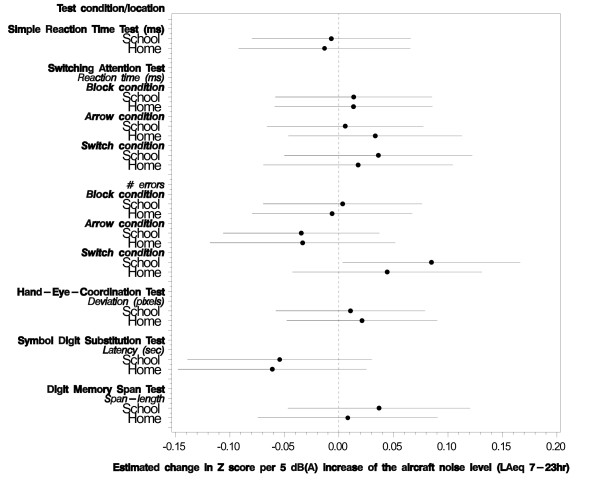
**The relation between aircraft noise exposure (L_Aeq, 7-23 hrs_) and the scores on the NES tests, adjusted for confounders**. The dotted vertical line corresponds to no effect of aircraft noise exposure. The circles correspond to the estimated change in Z-score per 5 dB(A) increase of the aircraft noise level and the horizontal lines correspond to the 95% confidence interval.

Figure 3 shows the fully adjusted associations between aircraft noise exposure at school and at home and the different scores of the NES tests. In order to be able to present the outcomes of the multilevel analyses in one figure, z-scores were computed. The results of the multilevel analysis show a statistically significant relation between aircraft noise exposure at school and the number of errors for the Switch condition of the SAT (χ^2 ^= 4.7, df = 1, p = 0.03): an increase of 0.96 (95%CI = 0.04 - 1.89) errors was found as aircraft noise exposure increased 10 dB(A) (see also Additional file 2, Table S2). Potential confounders that had a significant effect on the score of the SAT were parental support (children who received more parental support made more errors in the block condition), gender (boys made more errors in the arrow condition), mother's education (children of parents with a lower level of education made more errors in the switching condition), and main language spoken at home (children whose main language at home was Dutch, made less errors during the switching condition). Apart from the association found on the switch condition of the SAT, none of the cognitive outcomes measured by the NES were related to aircraft noise exposure at school. None of the cognitive outcomes measured by the NES were related to aircraft noise exposure at home. The effects of aircraft noise exposure did not change after exclusion of children suffering from ADHD and/or dyslexia.

**Figure 4:**
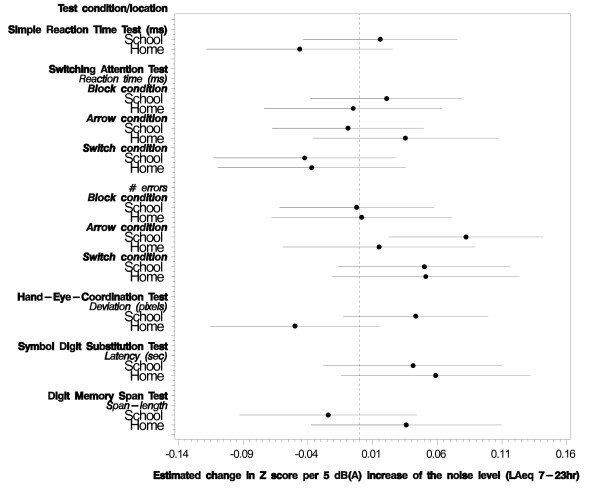
**The relation between road traffic noise exposure (L_Aeq, 7-23 hrs_) and the scores on the NES tests adjusted for confounders**. The dotted vertical line corresponds to no effect of road traffic noise exposure. The circles correspond to the estimated change in Z-score per 5 dB(A) increase of the road traffic noise level and the horizontal lines correspond to the 95% confidence interval.

Figure 4 shows the fully adjusted associations between road traffic noise exposure at school and at home and the different scores of the NES tests. Only the relation between road traffic noise at school and the number of errors during the arrow-condition of the SAT was statistically significant (χ^2 ^= 8.2, df = 1, p = 0.004). A 10 dB(A) increase in road traffic noise at school resulted in an increase of 0.27 (95%CI = 0.08 - 0.46) errors (see also additional file 2, Table S2). None of the cognitive outcomes measured by the NES were related to road traffic noise exposure at home.

## Discussion

In this study investigating the neurobehavioral effects of road traffic and aircraft noise exposure in 553 primary schoolchildren living around Schiphol Amsterdam Airport, effects of school noise exposure were observed in the more difficult parts of the SAT: children attending schools with higher road or aircraft noise levels made more errors. This is in agreement with the results of recent other studies investigating the effects of transportation noise exposure at school on children's cognitive functioning. In the Munich Airport Study, Evans and colleagues (1995) found that children from noise exposed communities had more errors on a difficult subscale of German standardized reading test than children from quiet communities; the two groups did not differ on the easy and intermediate portions of the test [14]. Meis and colleagues (1998) found similar adverse impacts on more complex memory tasks after comparing simulated and actual aircraft noise exposure in the lab and in the field [44]. In the West London Schools Study no significant difference on the score of the reading comprehension test was found between children in the noise and quiet groups. However, when the 15 most difficult items of the reading test were analyzed separately, a significant difference was found between the two noise exposure conditions [45]. From this study and previous scientific literature [46] it can be concluded that performance on simple tasks is less susceptible to the effects of noise than performance on more complex tasks, requesting increased mental performance. Since the NES was not administered in other studies investigating the effects of transportation noise exposure on children, a direct comparison was not possible. However, because of their consistency with the results of other studies investigating the effects of transportation noise on the more complex and difficult parts of cognitive tests, the results point to the conclusion that exposure to aircraft noise exposure impairs children's performance mainly on the difficult tasks.

By combining the NES with paper-and-pencil tests we were able to investigate the external validity of the NES. Compared to the paper-and-pencil tests, the coherence between the different NES tests was relatively high: two interpretable components could be derived. This supports the structure of the association between the separate NES-tests. From our results it can be concluded that the tests of the NES can complement the paper-and-pencil tests when investigating the effects of noise on children's cognitive functioning: in addition to the paper-and-pencil tests, the tests of the NES measure some different aspects of attention: Response speed and the ability to switch between responses.

From the literature it is known that computer-administered testing such as the NES, offers some advantages in comparison with paper-and-pencil testing: (i) NES is a standardized method that gives more test-leader independent results reducing observer bias; (ii) data collection by means of the NES is highly efficient: Both the administration, data handling and reporting of results are easy; (iii) in comparison with paper-and-pencil tests, the presentation of the test material is more consistent and responses are exactly timed; and (iv) furthermore, the computerized performance tests were well-accepted by the children; the game character of the tests usually stimulates motivation [32,47]. Computer-administered testing techniques also offer some disadvantages which might affect the test outcome: examinees may become frustrated because they cannot backtrack during computer testing. In addition, there is increased attention focused on individual items when they are presented singly during computer testing. Unfortunately, we were not able to test whether and how this has affected our outcomes.

Our study found an effect of noise exposure at school on the SAT, measuring the ability to switch between responses. No effects of home noise exposure were found. It is possible that exposure at school or home differently affected the outcomes of the switching attention test. In addition, it is possible that exposure to noise at home may have affected the outcomes of the tests by interacting with exposure at school. Such effects were already found in relation to annoyance where analyses indicated a carryover effect: children in high aircraft noise areas report more annoyance from aircraft noise in high road traffic noise areas than children in low road traffic noise areas and vice versa [48]. Unfortunately, these hypotheses can not be further investigated on the data available in the RANCH study, because of the substantial co-linearity between school and home noise exposure. And since the main objective of RANCH was to investigate the effects of noise exposure at school on children's cognition, noise exposure at home was not taken into account during the selection of the participants.

This study represents an improvement on previous studies due to its comprehensive inclusion of potential confounders and determinants. The hierarchical structure of the data (children within schools) has been taken into account, which was not the case in analyses of previous studies. The participants were distributed over a broad noise exposure range, and a continuous noise exposure measure was used in the statistical analysis, adding to the statistical power of the study. Most studies investigating the impact of noise exposure have involved between-group comparisons (high versus low): results of these studies may be sensitive to exposure-misclassification. The current study investigated the effects of both school and home noise exposure. Due to the high correlation between the noise metrics, it was not possible to disentangle the effects of school and home noise exposure. Another limitation of the study is its design and the lack of adjustment for special educational needs other then ADHD and dyslexia. Furthermore, the estimation of exposure to road traffic noise remains problematic: during their time at school, road traffic noise exposure changes as children move to a different classroom each year. Thus, the road traffic noise levels at the façade of their current classroom might not reflect the average level of exposure during their time at school. An additional problem is that road traffic noise exposure is less uniformly distributed across classrooms as air traffic noise. Although the NES was administered in a quiet room in the school, it was not possible to adjust for noises from any unexpected sources apart from aircraft and road traffic noise, which may have distracted the children and affected their concentration during the test. For the cognitive outcomes measured by the paper-and-pencil tests it was possible to adjust for noise from unexpected sources; after additional adjustment for these unexpected noises, the effects of road and air traffic noise did not change [6].

In this study statistically significant associations were observed between noise exposure at school (L_Aeq, 7-23 hrs_) and the child's' ability to switch between responses: as noise exposure levels increased, the number of errors made during the SAT increased. Road- and aircraft noise exposure was not associated with the other cognitive outcomes measured. However, it is difficult to indicate what our findings mean. The elevations in the number of errors found in relation to noise exposure were small and the clinical significance of such minor changes is difficult to determine. Because the effects of noise on many different cognitive outcomes were investigated, our findings could be the result of chance. However, one has to keep in mind that our findings are consistent with the literature; we have found an effect on the NES-outcomes where we expected to find an effect.

Due to the cross-sectional design of this study, it is unknown whether the neurobehavioral effects of noise are reversible if exposure to noise ceases; in the Munich Airport study differences in reading score between the two exposure groups disappeared after removing the differences in noise exposure [15]. The individual tests that are included into the NES reflect the concerted action of many neurobehavioral mechanisms or brain systems affected [49]. It can be concluded that in addition to cognition (having to do with the ability to think and reason), neurobehavioral components (having to do with the way the brain affects emotion, behaviour and learning) play also a role in the relationship with noise.

Currently, there is increasing attention for the possible cognitive effects of air pollution [50-54]. In 2008, the first epidemiological study investigating the effects of air pollution on children's cognitive functioning was presented [51]: the long-term concentration of black carbon particles from mobile sources was associated with decreases in cognitive test scores among 202 primary schoolchildren living in Boston. It is hypothesized that particles move to the brain tissue where they might cause oxidative stress and inflammatory reactions. Since children in urban areas often are exposed to several environmental exposures simultaneously, it is possible that the associations found in our study could also be attributed to traffic-related air pollution and not to road traffic and aircraft noise exposure; conversely, the effects found in the studies investigating the relation between air pollution and cognitive functioning could also be attributed to noise exposure. More research is necessary to disentangle the effects of traffic-related air pollution and noise exposure. Unfortunately, when writing this article, no data on exposure to traffic-related air pollution for the participating children were available.

## Conclusions

Based on these analyses the authors conclude that neurobehavioral tests can complement paper-and-pencil tests when investigating the effects of noise on children's cognitive functioning. PCA demonstrated that in addition to commonly used paper-and-pencil tests, neurobehavioral tests measure some different aspects of attention: Response speed and the ability to switch between responses. Effects of school noise exposure were observed in the more difficult parts of the SAT. Based on this study and previous scientific literature it can be concluded that performance on simple tasks is less susceptible to the effects of noise than performance on more complex tasks. It is not possible to draw definite conclusions about the relative importance of noise exposure at home and at school and possible interactions.

## Abbreviations

ADHD: Attention-Deficit Hyperactivity Disorder; CRIE: CITO Readability Index for Elementary and Special Education; DMST: Digit Memory Span Test; HECT: Hand-Eye Coordination Test; L_Aeq7-23 hrs_: Equivalent sound level from 7:00 to 23:00 hours; NES: Neurobehavioral Evaluation System; RANCH: Road Traffic and Aircraft Noise Exposure and Children's Cognition and Health: Exposure-Effect Relationships and Combined Effects; SAT: Switching Attention Test; SDST: Symbol-Digit Substitution Test; SRTT: Simple Reaction Time Test.

## Competing interests

The authors declare that they have no competing interests.

## Authors' contributions

EvK participated in the design of the study, collected the data, performed the statistical analyses and drafted the manuscript, IvK participated in the design of the study and helped to draft the manuscript, EL helped to draft the manuscript, JL and HE participated in the design of the study, collected the data, and helped to draft the manuscript. SS coordinated the RANCH project, participated in the design of the study, and helped to draft the manuscript. All authors have read and given final approval of the version to be published.

## Supplementary Material

Additional file 1 **Field studies investigating the effects of environmental noise exposure on the cognitive functioning of primary schoolchildren**. Table summarizing the main characteristics of studies that investigated the effects of environmental noise exposure on the cognitive performance of primary schoolchildren. Click here for file

Additional file 2 **The fully adjusted multilevel models for noise exposure at school and at home and the switching attention test (errors)**. Table presenting the multilevel models used for road- and aircraft noise exposure at school and at home and the errors made during the three conditions of the switching attention test. Click here for file

## References

[B1] World Health Organisation Website Noise and Health 2007 http://www.euro.who.int/en/what-we-do/health-topics/environmental-health/noise/facts-and-figures Website visited April 2007

[B2] Staatsen BAM Nijland HA van Kempen EMM de Hollander AEM Franssen EAM van Kamp I Assessment of health impacts and policy options in relation to transport related noise exposure. Topic paper noise 2004 The Netherlands: National Institute for Public Health and the Environment RIVM-report no. 81512000215469572

[B3] World Health Organisation Berglund B, Lindvall T, Schwela D, Koh KT Guidelines for community noise 2000 Geneva, Switzerland World Health Organisation

[B4] Knol AB Staatsen BAM Trends in the environmental burden of disease in the Netherlands 1980-2020 2005 Bilthoven: National Institute for Public Health and the Environment RIVM report no 500029001

[B5] van den Hazel P Zuurbier M (eds) PINCHE project: Final report WP1 exposure assessment 2005 Public Health Services Gelderland Midden, Arnhem, The Netherlands

[B6] Stansfeld SA Berglund B Clark C Lopez-Barrio I Fischer P Ohrstrom E Haines MM Head J Hygge S Kamp van I Berry BF on behalf of the RANCH study team Aircraft and road traffic noise and children's cognition and health: a cross-national study Lancet 2005 365 1942 1949 10.1016/S0140-6736(05)66660-3 15936421

[B7] Stansfeld S Haines M Brown B Noise and health in the urban environment Rev Environ Health 2000 15 43 82 10939085 10.1515/reveh.2000.15.1-2.43

[B8] Bronzaft AL The effect of a noise abatement program on reading ability J Environ Psychol 1981 1 215 222 10.1016/S0272-4944(81)80040-0

[B9] Green KB Pasternack BS Shore RE Effects of aircraft noise on reading ability of school-age children Arch Environ Health 1982 37 24 31 7059228

[B10] Cohen S Evans GW Krantz DS Stokols D Physiological motivational and cognitive effects of aircraft noise on children: moving from the laboratory to the field Am Psychol 1980 35 231 243 10.1037/0003-066X.35.3.231 7377650

[B11] Cohen S Evans GW Krantz DS Stokols D Kelly S Aircraft noise and children: longitudinal and cross-sectional evidence on adaptation to noise and the effectiveness of noise-abatement J Pers Soc Psychol 1981 40 331 345 10.1037/0022-3514.40.2.331

[B12] Cohen S Evans GW Stokols D Krantz DS Behaviour, health and environmental stress 1986 New York and London: Plenum Press

[B13] Sanz SA Garcia AM Garcia A Road traffic noise around schools: a risk for pupil's performance? Int Arch Occup Environ Health 1993 65 205 207 10.1007/BF00381157 8282419

[B14] Evans GW Hygge S Bullinger M Chronic noise and psychological stress Psychol Sci 1995 6 333 338 10.1111/j.1467-9280.1995.tb00522.x

[B15] Evans GW Bullinger M Hygge S Chronic noise exposure and physiological response: a prospective study of children living under environmental stress Psychol Sci 1998 9 75 77 10.1111/1467-9280.00014

[B16] Hygge S Evans GW Bullinger MA Prospective study on some effects of aircraft noise on cognitive performance in school children Psychol Sci 2002 13 469 474 10.1111/1467-9280.00483 12219816

[B17] Haines MM Stansfeld DA Job RFS Berglund B Head J Chronic aircraft noise exposure, stress responses, mental health and cognitive performance in school children Psychol Med 2001 31 265 277 11232914 10.1017/s0033291701003282

[B18] Haines MM Stansfeld SA Job RFS Berglund B Head J A follow-up of chronic aircraft noise exposure on child stress responses and cognition Int J Epidemiol 2001 30 839 845 10.1093/ije/30.4.839 11511614

[B19] Haines MM Stansfeld SA Brentnall S Head J Berry B Jiggins M Hygge S The West-London School Study: the effects of chronic aircraft noise exposure on child health Psychol Med 2001 31 1385 1396 11722153 10.1017/s003329170100469x

[B20] Haines MM Stansfeld SA Head J Job RFS Multilevel modeling of aircraft noise on performance tests in schools around Heathrow Airport London J Epidemiol Community Health 2002 56 139 144 10.1136/jech.56.2.139 11812814 PMC1732072

[B21] Lercher P Evans GW Meis M Ambient noise and cognitive processes among primary schoolchildren Environ Behav 2003 35 725 735 10.1177/0013916503256260

[B22] Hiramatsu K Tokyama T Matsui T Miyakita T Osada Y Yamamoto T de Jong RG, Houtgast T, Franssen EAM, Hofman WF The Okinawa study: effect of chronic aircraft noise exposure on memory of school children Proceedings of the 8th International Congress on Noise as a Public Health Problem 2003 Schiedam, The Netherlands: Foundation ICBEN 179 80

[B23] Shield BM Dockrell JE The effects of environmental and classroom noise on the academic attainments of primary school children J Accoust Soc Am 2008 123 133 144 10.1121/1.2812596 18177145

[B24] Beaman CP Auditory distraction from low-intensity noise: a review of the consequences for learning and workplace environments Appl Cognit Psychol 2005 19 1041 1064 10.1002/acp.1134

[B25] Xue J McCurdy Th Sprengler J Özkaynek H Understanding variability in time spent in selected locations for 7-12 year old children J Expo Anal Environ Epidemiol 2004 14 222 233 10.1038/sj.jea.7500319 15141151

[B26] Freijer JL de Loos S Activity patterns of the Dutch population to support the development of a system of dosimetrics for exposure to noise 1998 Bilthoven: National Institute of Public Health and the Environment RIVM report 715120002

[B27] Maquet P The role of sleep in learning and memory Science 2001 294 1048 1052 10.1126/science.1062856 11691982

[B28] Richardson DB Loomis D The impact of exposure categorization for grouped analyses of cohort data Occup Environ Med 2004 61 930 935 10.1136/oem.2004.014159 15477287 PMC1757839

[B29] Bearer CF The special and unique vulnerability of children to environmental hazards Neuro Toxicology 2000 21 925 934 11233762

[B30] Emmen HH Staatsen BAM Deijen JB A feasibility study of the application of neurobehavioural tests for studying the effects of aircraft noise on primary schoolchildren living in the vicinity of Schiphol Airport the Netherlands 1997 Bilthoven: RIVM Report no 441520007

[B31] Baker EL Letz R Neurobehavioral testing in monitoring hazardous workplace exposures J Occup Med 1986 28 987 990 10.1097/00043764-198610000-00017 3534164

[B32] Letz R Use of computerized test batteries for quantifying neurobehavioural outcomes Environ Health Perspect 1991 90 195 198 10.2307/3430868 2050061 PMC1519520

[B33] Emmen HH Hoogendijk EMG Hooisma J Orlebeke JF Uijtdehaage SHJ Adaptation of two standardized international test batteries for use in the Netherlands for detection of exposure to neurotoxic compounds 1988 Rijswijk, The Netherlands: TNO Medical Biological Laboratory Internal report 1988-18

[B34] Clark C Martin R van Kempen E Alfred T Head J Davies HW Haines MM Lopez-Barrio I Matheson M Stansfeld SA Exposure-effect relations between aircraft and road traffic noise exposure at school and reading comprehension. The RANCH-project Am J Epidem 2006 163 27 37 10.1093/aje/kwj001 16306314

[B35] Dassen AGM Jabben J Dolmans JHJ Development and use of EMPARA: a model for analysing the extent and effects of local environmental problems in the Netherlands Proceedings of the 2001 International Congress and Exhibition on Noise Control Engineering 2001 The Hague, The Netherlands: Acoustical Society of The Netherlands

[B36] Arcia E Otto DA Reliability of selected tests from the Neurobehavioral Evaluation System Neurotoxicol Teratol 1992 14 103 110 10.1016/0892-0362(92)90058-I 1593984

[B37] Staphorsius G Leesbaarheid en leesvaardigheid De ontwikkeling van een domeingericht meetinstrument (Thesis) 1994 Arnhem: Cito ISBN nr: 90-801795-2-3

[B38] Cohen MJ Children's Memory Scale Manual 1997 The Psychological Corporation Harcourt Brace and Company: San Antonion. TX

[B39] Smith AP Miles C The combined effects of occupational health hazards: an experimental investigation of the effects of noise, nightwork and meals Int Arch Occup Environ Health 1987 59 83 89 10.1007/BF00377682 3793248

[B40] Hygge S Boman E Enmarker I The effects of road traffic noise and meaningful irrelevant speech on different memory systems Scand J Psychol 2003 44 13 21 10.1111/1467-9450.00316 12602999

[B41] Toulouse E Pieron H Toulouse-Pieron Prueba Perceptiva y de Atencion. [in Spanish] (Test of perception and attention) 1986 Madrid: TEA

[B42] Stansfeld S Haines M Matheson M Asker R Davison C Klineberg E Psychometric pilot study report of RANCH cognitive tests 2002 London: University of London QMUL

[B43] Costello AB Osborn JW Best practices in exploratory factor analysis: four recommendations for getting the most from your analysis Practical Assessment Research & Evaluation 2005 10 http://pareonline.net/getvn.asp?v=10&n=7

[B44] Meis M Hygge S Evans GW Bullinger M Effects of traffic noise on implicit and explicit memory: results from field and laboratory studies Proceedings of 7th the International Congres on Noise as a Public Health Problem Sydney Noise Effects 98 Ltd 1998

[B45] Matheson MP Stansfeld SA Haines MM The effects of chronic aircraft noise exposure on children's cognition and health: 3 field studies Noise Health 2003 5 31 40 12804210

[B46] Park JF Payne MC Jr Effects of noise level and difficulty of task in performing division J Appl Psychol 1963 47 367 368 10.1037/h0048773

[B47] Emmen HH Staatsen BAM Deijen JB Fischer PH Van Kamp I Neurobehavioral measurements in children living around Schiphol Airport. Further methodological considerations Proceedings of the 2001 International Congress and Exhibition on Noise Control Engineering 2001 The Hague, The Netherlands: Acoustical Society of The Netherlands

[B48] van Kamp I Nilsson ME van Kempen E Stellato RK Lopez-Barrio I Stansfeld S Combined effects of aircraft noise and road traffic noise on annoyance: the RANCH study Proceedings of the 33rd International Congress and Exhibition on Noise Control Engineering. Prague:22-25 August 2004

[B49] Anger WK Neurobehavioral tests and systems to assess neurotoxic exposures in the workplace and the community Occup Environ Med 2003 60 531 538 10.1136/oem.60.7.531 12819291 PMC1740574

[B50] Crüts B van Etten L Törnqvist H Blomberg A Sandström T Mills NL Borm PJA Exposure to diesel exhaust induces changes in EEG in human volunteers Part Fibre Toxicol 2008 5 4 10.1186/1743-8977-5-4 18334019 PMC2329662

[B51] Suglia SF Gryparis A Wright RO Schwartz J Wright RJ Association of black carbon with cognition among children in a prospective birth cohort study Am J Epidemiol 2008 167 280 286 10.1093/aje/kwm308 18006900

[B52] Chen JC Schwartz J Neurobehavioural effects of ambient air pollution on cognitive performance in US adults Neuro Toxicology 2009 30 231 239 10.1016/j.neuro.2008.12.011 19150462

[B53] Perera FP Li Z Whyatt R Hoepner L Wang S Camann D Rauh V Prenatal airborne polycyclic aromatic hydrocarbon exposure and child IQ at age 5 years Pediatrics 2009 124 195 202 10.1542/peds.2008-3506 PMC2864932 19620194

[B54] Wang S Zhang J Zeng X Zeng Y Wang S Chen S (2009) Association of traffic-related air pollution with children's neurobehavioral functions in Quanzhou, China Environ Health Perspect 2009 10.1289/ehp.0800023 PMC2790518 20019914

