# Supplementary material for: Neurobehavioral effects of transportation noise in primary schoolchildren: a cross-sectional study
Source: Environ Health. 2010 Jun 1;9:25. doi: 10.1186/1476-069X-9-25 (PMC2898757; doi:10.1186/1476-069X-9-25)
Supplement: Additional file 1 — Field studies investigating the effects of environmental noise exposure on the cognitive functioning of primary schoolchildren. Table summarizing the main characteristics of studies that investigated the effects of environmental noise exposure on the cognitive performance of primary schoolchildren. [file 1476-069X-9-25-S1.PDF]

**Additional file 1**

**Table S1 – Field studies investigating the effects of environmental noise exposure on the cognitive functioning of primary schoolchildren.**

| **Study** | **Location**[*)] | **Design**[†)] | **Schools (n)** | **Children** | | **Exposure** | | **Cognition** | |
|---|---|---|---|---|---|---|---|---|---|
| | | | | **N** | **Age (yr)** | **Source**[‡)] | **Noise indicator**[$)] | **Ability tested**[#)] | **Test used**[**)] |
| Bronzaft [8] | NY, US | FU | 1 | 955 | 7-11 | T | Quiet vs. Noisy | 1 | CAT |
| Green [9] | NY, US | Ec | 362 | 8240 | 7-11 | A | | 1 | National Standardized Tests of Reading ability |
| LAS [10-12] | LA, US | CS, FU | 7 | 262 | 8-9 | A | Peak level | 1, 2<br>3,4<br><br>5 | CTBS<br>Cross-out letters in ambient or distracting condition<br>WAD |
| Sanz [13] | Valencia, Sp | CS | 2 | 136 | 6-11 | R | Mean equivalent levels | 4 | Faces test and crossing-out 2 letters from a text |
| MAS [14-16] | Munich, Ger | BA | - | 326 | 9-10 | A | $L_{Aeq, 24hr}$ | 1<br>6<br>7, 8 | Standardized German Reading test<br>Recall of a test<br>Backward recall of consonants |

| | | | | | | | | | |
|---|---|---|---|---|---|---|---|---|---|
| | | | | | | | | 4<br>9 | Visual search task and<br>Reaction time test |
| SEHSa [17] | London,<br>UK | CS | 8 | 340 | 8-11 | A | $L_{Aeq, 16hr}$ | 10<br>6<br>8 | SRS<br>Recognition and recall<br>Serial-digit recall task |
| SEHSb [18] | London,<br>UK | FU | 8 | 275 | 8-11 | A | $L_{Aeq, 16hr}$ | 10<br>11 | SRS<br>TEA-Ch, scoring activity |
| WLSS [19] | London,<br>UK | CS | 20 | 451 | 8-9 | A | $L_{Aeq, 16hr}$ | 10<br>12, 13, 14<br>7<br>11 | SRS-2<br>CMS, story C & D<br>Backward digit recall<br>TEA-Ch, Score activity |
| SATS [20] | London,<br>UK | Ec | 123 | 11,000 | 11 | A | $L_{Aeq, 16hr}$ | 2, 15 and 16 | SATs, Key Stage 2 tests |
| TMS [21] | Inn<br>Valley,<br>Au | CS | 26 | 1230 | 8-11 | T, R | $L_{dn}$ | 17, 18<br>4 | LMT<br>Visual search task |
| Okinawa [22] | Okinawa, | CS | 11 | 2269 | 8-11 | MA | $L_{dn}$ | 6, 8 | Auditory and visual short-term |

| | | | | | | | | | |
|---|---|---|---|---|---|---|---|---|---|
| | Jp | | | | | | | | memory tests |
| Shield [23] | London, UK | Ec | 142 | | 7 and 11 | E, I | $L_{Aeq}$, $L_{A90}$ and $L_{Amax}$ | 1, 2, 15, 19 and 20 | SATs, Key Stage 1 tests<br>SATs, Key Stage 2 tests |

*) NY = New York, US = United States, LA = Los Angeles, Sp = Spain, Ger = Germany UK = United Kingdom, Au = Austria, Jp = Japan; †) CS = cross sectional study, FU = Follow/up study, Ec = Ecologic study, BA = Before-after study; ‡) T = Train, A = Aircraft, R = Road traffic, MA = Military Aircraft, E = External noise, I = Internal noise; $) $L_{Aeq,\ 24hr}$ = The equivalent continuous sound level over 24 hours, $L_{Aeq,\ 16hr}$ = The equivalent continuous sound level over 16 hours, $L_{dn}$ = Day-night sound level, the average noise level over a 24 hour period, $L_{Aeq}$ = Equivalent sound pressure level in dB(A), $L_{90}$ = The sound level exceeded for 90% of the time, used to describe the background noise level, $L_{Amax}$ = the maximum level of sound, i.e. the peak level of sound measured in any given period; #) Cognitive ability tested: 1 = Reading, 2 = Mathematics, 3 = Incidental Memory, 4 = Attention, 5 = Auditory Discrimination, 6 = Long-term Memory, 7 = Working memory, 8 = Short-term memory, 9 = Speech perception, 10 = Reading comprehension, 11 = Sustained Attention, 12 = Memory, immediate recall, 13 = Memory, delayed recall, 14 = Memory, delayed recognition, 15 = Science, 16 = English, 17 = Intentional Memory, 18 = Incidental memory, 19 = Writing, 20 = Spelling; **) CAT = California Achievement Test, CTBS = California Test of Basic Skills, WAD = Wepman Auditory Discrimination Test, Faces test = Difference Perception Test, SRS = Suffolk Reading Scale, TEA-Ch = Tests of Everyday Attention for Children, CMS = Child Memory Scale, SAT = Standard Assessment Task, LMT = Lern und Merkfähigkeitstests
