# Supplementary material for: Neurobehavioral effects of transportation noise in primary schoolchildren: a cross-sectional study
Source: Environ Health. 2010 Jun 1;9:25. doi: 10.1186/1476-069X-9-25 (PMC2898757; doi:10.1186/1476-069X-9-25)
Supplement: Additional file 2 — The fully adjusted multilevel models for noise exposure at school and at home and the switching attention test (errors). Table presenting the multilevel models used for road- and aircraft noise exposure at school and at home and the errors made during the three conditions of the switching attention test. [file 1476-069X-9-25-S2.PDF]

Additional file 2

Table S2 - The fully adjusted multilevel models for noise exposure at school and at home and the switching attention test (errors)

Outcome →	Block_f		Arrow_f		Switch_f	
	B (SE)	B(SE)	B (SE)	B (SE)	B (SE)	B (SE)
Fixed coefficients ↓						
Intercept	1.713 (1.008)	1.687 (0.986)	2.604 (1.574)	2.848 (1.645)	10.098 (5.946)	11.725 (5.737)
Aircraft noise at school	0.001 (0.008)		-0.011 (0.012)		0.097 (0.047)*	
Road traffic noise at school	0.000 (0.006)		0.026 (0.010)*		0.057 (0.039)	
Aircraft noise at home		-0.001 (0.008)		-0.011 (0.014)		0.050 (0.046)
Road traffic noise at home		0.000 (0.007)		0.005 (0.012)		0.058 (0.042)
Age (yrs)	-0.176 (0.093)	-0.154 (0.092)	-0.170 (0.146)	-0.065 (0.150)	-0.515 (0.536)	-0.462 (0.531)
Boys	0.157 (0.099)	0.158 (0.099)	0.398 (0.154)*	0.388 (0.156)*	0.438 (0.556)	0.435 (0.560)
Employed	0.031 (0.186)	-0.014 (0.191)	-0.549 (0.290)	-0.532 (0.300)	-0.188 (1.045)	-0.183 (1.078)
Crowded	-0.175 (0.105)	-0.179 (0.106)	-0.095 (0.164)	-0.103 (0.167)	-0.421 (0.593)	-0.441 (0.600)
Home owner	0.008 (0.130)	-0.004 (0.132)	0.082 (0.203)	0.077 (0.210)	0.749 (0.743)	0.588 (0.750)
Mother's education	0.094 (0.182)	0.100 (0.184)	0.247 (0.285)	0.202 (0.291)	2.176 (1.033)*	2.187 (1.042)*
Main language is Dutch	0.120 (0.236)	0.123 (0.233)	0.003 (0.368)	-0.065 (0.384)	-2.849 (1.368)*	-2.395 (1.350)
Long standing illness	0.118 (0.110)	0.116 (0.111)	0.082 (0.172)	0.077 (0.174)	0.624 (0.620)	0.720 (0.626)
Parental support	0.071 (0.026)*	0.071 (0.027)*	-0.006 (0.041)	0.003 (0.042)	-0.195 (0.148)	-0.176 (0.150)

Classroom glazing						
Single	0.089 (0.308)		0.237 (0.481)		1.167 (1.815)	
Double	0.125 (0.308)		0.430 (0.481)		0.735 (1.812)	
Triple	Ref		Ref		Ref	
Double glazing at home		0.020 (0.098)		0.000 (0.156)		0.737 (0.559)
Random Parameters						
Level 2: School	0.000	0.000	0.000	0.0542	0.450	0.305
Level 1: Pupil	1.003	1.009	2.445	2.467	31.660	32.069

B = Estimated change in the test score per dB(A); SE = Standard Error; N = sample size.
